# A leap towards enforcing medicines prescribing by generic names in low- and middle-income countries (LMICs): pitfalls, limitations, and recommendations for local drug regulatory agencies

**DOI:** 10.1186/s40545-022-00501-4

**Published:** 2022-12-22

**Authors:** Hamid A. Merchant, Zaheer-Ud-Din Babar, Izhar M. Hussain

**Affiliations:** 1 Department of Pharmacy, School of Applied Sciences, University of Huddersfield, Queensgate, Huddersfield, HD1 3DH UK; 2 Health Services Academy, Park Road, Chak Shahzad, Islamabad, 44000 Pakistan; 3 Institute of Business and Health Management (IBHM), Dow University of Health Sciences (DUHS), SUPARCO Road, Karachi, 74200 Pakistan

**Keywords:** DRAP, Prescribing, Low-cost generics, Access to medications, Bioequivalence, Biosimilars, Biowaivers, Low- and middle-income countries (LMICs)

## Abstract

The Drug Regulatory Authority of Pakistan (DRAP) in response to the public outcry on increasing medicines prices in the country issued notifications to direct healthcare professionals to prescribe medicines with their generic names. Like DRAP, many regulators in the low- and middle-income countries (LMICs) are also inspiring from the west to legally enforce generic prescribing in a bid to reduce the out-of-pocket public expenditures. However, there are pitfalls in the LMICs drug regulatory framework, which if left unaddressed can severely jeopardise the foreseen benefits of medicines prescribing by generic names. This article critically appraises the impact of prescribing by generic names regulations in LMICs and highlights the key considerations that are vital to address before legally enforcing generic prescribing. The ethics, regulatory compliance, and good governance are the key to success; better generics for a better tomorrow.

## Background

The Drug Regulatory Authority of Pakistan (DRAP) recently issued notifications directing all provincial governments with directives to prescribe medicines with their generic names in all public and private healthcare sector in the country [1, 2]. DRAP notification came in response to the public outcry on increasing medicines prices in the country. The pharmaceutical companies are often accused of offering financial incentives in encouraging prescribers to favour a particular brand. We welcome the new regulations that were issued in anticipation to reduce public out-of-pocket expenditure on medicines by increasing the public access to low-cost generic drugs. The DRAP has also sought stakeholder’s opinion on generic medicines registrations [3]. We, therefore, in this article, highlight the pitfalls of the newly proposed generic prescribing regulations, which if left unaddressed can jeopardise the foreseen benefits. Table 1 summarises the key recommendations to DRAP and other regulatory authorities in low- and middle-income countries (LMICs) before generic prescribing are legally enforced in their respective jurisdictions.

**Table 1:** Recommendations to the DRAP and drug regulatory agencies in various low- and middle-income countries

• Implementation of bioequivalence regulations to ensure the bioavailability and therapeutic efficacy of generics are at par with the innovator’s product
• Ensure compendial monograph compliance to all active (APIs) and inactive ingredients (excipients), and formulated products, where applicable
• Implement SUPAC (scale-up and post-approval changes) regulations to ensure that adequate regulatory controls are in place to assess the biopharmaceutic impact of post-approval changes in the product, its formulation, or changes in raw material or their suppliers, equipment or manufacturing processes, or facilities, etc.
• Special consideration for potent drugs, psychoactive substances, cytotoxic drugs, etc.
• Exceptional consideration for modified release or specially formulated products (e.g., ER, XR, CR, SR, DR etc.)
• Special considerations for biological drugs and implementation of biosimilars/biobetters regulations
• Allowance for consumer products (GSL and OTC products), polypills, etc.
• Considerations for medical devices, such as inhalers
• Pricing caps for generics to ensure that they are not registered above the registered price of an innovator’s brand if already available in the country
• Enforcing regulations that all prescription drugs are ONLY dispensed on the prescription of registered medical practitioner and must be dispensed by a licensed pharmacist
• Ensure that all drug stores in the country operate under the direct supervision and presence of registered pharmacists
• Encourage ethical prescribing by enforcing antibribery regulations and subjecting prescribers to ‘fitness to practice’ and ‘code of conduct’ investigations on report or suspicion of a conflict of interest
• Ethics, regulatory compliance, and good governance are the key to have ‘better generics’ for a ‘better tomorrow’

### What are generic medicines?

All medicines have an international non-propriety name, commonly referred to as ‘generic or chemical name’ in addition to a brand name that is proprietary to the pharmaceutical company. A good example is ‘paracetamol’ that is famously branded as Panadol and Calpol in different parts of the world. Consumers, pharmacists and prescribers often have different preferences for a brand, and similar to the brands in consumer goods industry, often branded medicines are expensive and often have a perceived impression of good quality among consumers and prescribers. The question often asked by the public and governmental organisations is whether the branded medicines are really any better than the cheaper generics or is it just a consumer or prescriber’s perception?

To bring this into perspective one must understand how new medicines are discovered and how their generics are introduced. The innovator companies invest millions in drug discovery to bring new molecules from the laboratory and preclinical evaluations in animal models through to the clinical trials. It usually takes about 10 years to get a new drug to the market, costing millions of dollars to meet the toughest safety and efficacy standards imposed by the drug regulatory authorities. A stark reality is that majority of the new drugs and molecules do fail during their journey and never make it to the market. This highly risky investment in pharmaceuticals rarely produces a successful candidate that reaches the market. This new medicine then becomes the intellectual property of the innovator company who in return enjoys the sole right to manufacture and sell the new drug product throughout its patent life, which usually lasts for 20 years. However, to protect the return on investments, often drugs are patented very early in the discovery stage, therefore, by the time a product is launched in the market, there is often a fraction of the patent life left for the company to get the return of their extensive research and product development costs. Once patents are expired, other companies may then start producing the same product, often referred to as generics. The drug regulatory authorities, like FDA or EMA in their respective jurisdictions, ensure that these low-cost alternatives (aka generics) meet the same standards of quality (similar safety and efficacy) as of innovator’s product. These generics are priced much lower than the innovator’s product, as these generic manufacturers do not invent new drugs, and their costs are mainly attributed to standard manufacturing operations. Generics are always given preference, wherever possible, in countries like United Kingdom to reduce the cost of therapy and burden on national health services.

### Consumers, and healthcare professionals’ perception of generics

The research has shown that consumers [4–6] and healthcare professionals [7–10] often have several misconceptions about generic medicines across a spectrum of low-, middle- and high-income countries [11, 12]. It is, however, acknowledged that consumers acceptance of generics largely depends on pharmacists and prescribers’ recommendations. Over the years, patients’ and prescribers’ confidence and knowledge on generic medicines have improved, particularly in the developed world, however, more work is needed in LMICs. The lack of the acceptability of generics could lead to non-concordance (therefore, treatment failure) and is counterintuitive on treatment costs [13]. Studies have indicated that interventions [14] such as education, financial incentives, and better communication between patients and healthcare professionals has improved the confidence on generics.

So, if innovator brands are expensive and generic products provide a low-cost alternative, then it should make sense that the DRAP has decided to direct all clinicians in Pakistan to prescribe medicines by their generic names. It is true that in many countries, for instance in United Kingdom, generic prescriptions are a norm in the general medical practice but there are serious issues that needs to be considered when it comes to legally enforcing generic prescribing in Pakistan. This is also common across a spectrum of LMICs.

### Pitfalls of the law enforcing generic prescribing in Pakistan and other low- and middle-income countries (LMICs)

#### Lack of evidence on bioequivalence and lack of compendial compliance

The generics must demonstrate that the low-cost product is equivalent to the innovator’s product in its safety and efficacy, which is often demonstrated by ‘bioequivalence’ studies. The established regulatory authorities such as United States’ Food and Drug Administration (FDA) [15, 16], UK’s Medicines and Healthcare products Regulatory Agency (MHRA) [17] or the European Medicines Agency (EMA) [18] in the Europe issues marketing authorisation for generic products in their respective markets after vigorous scrutiny of generic products quality. In Pakistan, like many low-middle-income countries, the generic products are approved without demonstrating bioequivalence or going through the tight scrutiny that generic medicinal products must go through to get access to the western markets.

All medicines and each manufactured batch thereof must meet the relevant pharmacopeial specifications in developed countries, like UK or the USA. Pharmacopoeias are the gold standards of quality and contains monographs for pharmaceutical ingredients and finished products setting specifications for quality and manufacturing controls. Examples include the British Pharmacopoeia (BP) [19], European Pharmacopoeia (Ph.Eur.) [20], United States Pharmacopoeia (USP) [21]. In low- and middle-income countries including Pakistan, these compendial monographs are not legally enforced, and hence, the manufacturers do not need to demonstrate compendial compliance by law, but some may choose to do so as a mark for their quality or often because it’s a regulatory requirement in one of the markets where their products are exported.

In the absence of compendial compliance, often raw materials used in the manufacture of medicinal products are not of same quality as compendially compliant materials, and sourcing of those materials are also inconsistent. Also lack or even absence of quality assurance practices in material sourcing and vendor approval processes, and lack of stability studies mean that the finished products are not manufactured with consistent quality, and are subjected to increased inter- and intra-batch variability. Instead of adopting a comprehensive quality assurances system, over-reliance on finished product testing as a means of  controlling or assuring quality often results in  significant lapses leading to severe safety and efficacy issues.

The 2012 incident in Punjab Institute of Cardiology is an invaluable case study where hundreds of patients died due to a lethal contamination in heart medicines [22]. The recent 2022 incident in Gambia [23] is a reminder of Edwards Deming's famous quote “Quality cannot be tested; it is built into the product”. The Gambian incident and WHO alert caught the eyes of many regulatory agencies and, in particular, similar issues were discovered in India [24] and Indonesia [25] where hundreds of children died due to a number of substandard paediatric liquids that cannot be simply identified in the routine finished product testing by the quality control laboratories. This oversight has recently costed hundreds of innocent lives globally and reminds us of the importance of compendial compliance for all materials (APIs or excipients) to be used in the manufacture of medicinal products.

Similar to the SUPAC (scale-up and post approval changes) regulations in the United States, there are no formal regulations enforced in Pakistan or other LMICs to assure bioequivalence on SUPAC cases that may significantly impact the safety and efficacy of finished products. The regulatory authority in Pakistan (DRAP) and other LMICs are recommended to implement SUPAC regulations [26–28] to ensure that adequate regulatory controls are in place to risk assess the biopharmaceutic impact of post-approval changes in the product, its formulation, or changes in the raw material or their suppliers, equipment or manufacturing processes, or facilities, etc., before those changes are regularised in routine manufacture of medicinal products.

#### Safety considerations for potent drugs

Some drugs are classified as ‘potent’ drugs, or the drugs with ‘narrow therapeutic index’, these medicines are biologically active at a very small dose, such as in micrograms, and a very tight control of dose uniformity is essential to maintain efficacy and to prevent drug induced toxicity. Some examples include psychoactive and cytotoxic drugs. Subtle changes in the formulation and manufacturing methods may results in significant changes in product performance and safety for these substances. Generic medicines must meet a higher level of standards for these medicines to ensure the efficacy and safety is at par to the innovator’s product. The drug regulatory authority in Pakistan like various other LMICs have not been able to enforce these standards yet.

#### Modified release products

Some pharmaceutical products employ proprietary formulation strategies to optimise the release of medicinal agents from the product. These products are often referred to as ‘modified release’ (MR), ‘sustained release’(SR), ‘controlled release’(CR), ‘extended release’ (ER), or ‘timed-release’ products, etc. In contrast to the conventional products, the release of drug from these products is modified or controlled over an extended period of time using specialised formulation technologies. The efficacy and safety of these products, therefore, mainly relies on proprietary formulae (trade secrets) of the manufacturer. These products are unique in their characteristics and once patients are maintained on a particular product, it is not advised to switch to another product unless inevitable, for instance product discontinuation or shortage of supply. Switch to a different product, nevertheless, requires a close monitoring of the patient, often needing adjustment of dosing schedules, that may lead to tolerability, poor compliance, or adverse drug reactions. These products are usually preferred to be prescribed with their brand names even in the United Kingdom to ensure consistency in prescribing and dispensing for the safety of  patients. The generic copies of these products are also branded (local brands), and low-cost alternatives are also prescribed with their brand names as they cannot be exactly the same as the innovator’s product to ensure consistency and patients’ safety.

#### Biologicals

Unlike small drug molecules, the biological products such as insulin, vary significantly in their proprietary formulation strategies and not all insulin products are equivalent to each other when it comes to the rate and extent of insulin release from the injection site and consequent differences in blood sugar maintenance over a 24-h period. It takes hard work for the diabetologists and specialist diabetes nurses to monitor a new diabetic patient starting on insulin products to optimise a desired combination of immediately acting and prolonged-acting insulin formulations to personalise the dose for a patient. Once a patient is settled on a dose combination of insulin, the prescribers stick to the same branded combinations to ensure safety and compliance.

Unsolicited changes in dispensing these products are not safe and can lead to life threatening hypoglycaemic conditions. The approval process for the low-cost alternatives of the innovator’s biological products undergoes a much higher level of scrutiny under ‘biosimilar’ regulations before they are approved in western markets. The low-cost generics of biological products are also branded and are prescribed with their brand names to ensure consistency in clinical response and patients’ safety until a product-specific standardised biosimilar regulatory framework is introduced. The introduction of biobetters (where generics are made or designed even better than the innovators original products) makes it even more complex to standardize the prescribing practice.

#### Medical devices

The medical devices are often prescribed by brands, for example the inhalers for respiratory diseases. Inhalers are very specialised and precisely engineered medical devices that uses proprietary techniques to aerosolise very fine droplets or dry particles of the drugs to be delivered into the airways. There is a huge variation in the emitted dose between different products, device resistance and lung capacity of the patient. The selection of an appropriate inhaler requires remarkable efforts from practitioners to ensure that product selection is personalised to the patients need and patients are tutored on inhaler technique by specialist respiratory nurses. The inhalation technique also varies between different brands of the inhalers. Once patients are maintained on a particular type of inhalers, both the prescriber and pharmacist ensures that patient is always supplied with a similar device to ensure safety and compliance. Therefore, specialised medical devices, such as inhalers are also prescribed either with their brand names or extended generic names that are followed by the inhalation device type descriptors to ensure patients’ safety and compliance. It is, therefore, not straightforward for all inhalation devices to be simply registered and prescribed with their generic names only.

#### Polly pills

Several pharmaceutical products contain more than one medicinal agent, often referred to as combination products or simply ‘poly pills’. Common examples include products for diabetes, hypertension and combination therapies for infectious diseases such as tuberculosis. Often some patients with chronic illness, for instance diabetes and hypertension, must take several medicines in a day that often leads to poor adherence and compliance. Poly pills often addresses this problem and reduces the number of tablets or capsules that a patient otherwise take in a day. These polypills contains a unique combination of medicines and dosages, therefore, usually prescribed by their brand names to reduce dispensing errors and ensuring safety and compliance. Other examples may include multivitamins which are also branded to ensure consistency of dispensed formulation and patient’s compliance.

#### Locally branded products vs. innovators’ brands

It is important to understand that all branded medicinal products in Pakistan and other low- and middle-income countries are not the innovators brands. In Pakistan for instance, all locally produced generic medicinal products are also branded with proprietary trade names. These are often termed as’branded generics’. The industry prefers branded generics to ensure that all advertising and marketing efforts from a local pharmaceutical company could influence prescriber and/or consumer decision towards a particular product.

The irony is that often locally branded products in some countries may not always be cheaper than the innovator’s brands that are also locally available in the same market. It is not a general practice, but this discrepancy often occurs due to a very tightly regulated medicines pricing structure in some countries, for instance Pakistan. If innovators’ brands were registered decades ago and were registered with retail prices calculated based on then manufacturing costs (material, labour, utilities etc.). Once registered, if prices were not regularly revised on retail price index unlike general consumer goods where retail prices are regularly revised with inflation. The price for a recently registered generic product (a copy of the innovator’s brand registered many years ago) is based on the current manufacturing costs and, therefore, often end up being registered and sold at an equal or in some cases even at higher retail prices than the innovators original product. The drug regulatory agencies in the LMICs are therefore recommended to ensure pricing caps are in place to ensure that generics are not registered above the registered price of an innovator’s brand if already available in the same country.

#### Over-the-counter consumer products or general sales list items

Often branding is inevitable for the over-the-counter medications, for instance medications used for minor ailments, e.g., cough and cold or general-purpose pain relief medications, most of which are available to purchase over-the-counter without prescriptions. These products are often advertised in television, print or social media for influencing consumer behaviour. Branding these products also helps consumers to select a particular product that meets their personal taste for better concordance and compliance, for instance, specially formulated products to improve taste and palatability, size, or shape of the dosage forms or the choice of flavours etc.

#### Pharmacies run by unqualified personnel

The most drug stores in low- and middle-income countries including Pakistan are not like community pharmacies in the United Kingdom (or other developed countries) that are run by qualified pharmacists. Most shops retail selling (dispensing) medications in Pakistan, even in urban settings, are run by unqualified personnel (non-pharmacists), often not educated beyond a secondary school certificate. A new law enforcing generic prescribing might put patients at the mercy of those shopkeepers who may only stock products that favour a high profit margin for their business. It is very unlikely that these savings will be passed on to the end consumers; this will potentially open the doors for unwarranted monetary incentives towards distributors and retailers to manipulate the supply chain in favour of particular products. This will also put patients at the risk of receiving inappropriate products, in particular, where many products remain bioinequivalent to each other (the absence of bioequivalence regulations), this may risk patient safety for cases like potent drugs, modified-release products, medical devices, poly-pills, biologics, etc. as discussed earlier. The law enforcement agencies must ensure that pharmacies and drug stores are run by qualified and licensed pharmacist across the country [29].

#### The ethical dilemma

The core of the problem in Pakistan and other LMICs is poor ethics in the medical practice combined with a poor enforcement of antibribery regulations in these countries, where some pharmaceutical organisations solely rely on monetary incentives to medical practitioners in order to get their products prescribed. These incentives incudes a range of perks and discounts, free gifts, foreign holidays, luxury residential and office refurbishments, and even direct cash incentives.

The pharmaceutical companies have a huge marketing budget which they are legally allowed to spend on sales promotion activities, some good practice examples include promoting health education among public, health screening, public health promotion activities, medical education and supporting medical research etc. Often, majority of pharmaceutical companies in LMICs find it difficult to compete in a very crowded generics market, in particular, when they do not invest in innovative products. It then becomes very difficult for pharmaceutical companies to convince prescribers on scientific grounds to favour a particular product in their medical practice. This is when, the bribery and financial incentives help gaining a market share to achieve sales targets in a highly competitive and crowded pharmaceutical market in Pakistan and other LMICs.

The ethical and regulatory dynamics in the developing world are rapidly changing. The poor practices often perceived acceptable in the developing world today may no longer be acceptable tomorrow. In 2014, the CEO of a large pharmaceutical company was deported from China on pleading guilty of bribing doctors to prescribe their products. The pharmaceutical company was also fined millions of British pounds as a penalty for their illegal sale promotion practices in China [30, 31]. The ethics in professional practice should remain a fundamental part of medical and pharmacy education.

## Conclusions

Indeed, the generics have significantly reduced the expenditure on medicine and healthcare costs globally, and medicines prescribing by generic names has become a norm in many countries with well-established drug regulatory framework. However, there is substantial work required to improve the quality of generics in LMICs, and interventions to strengthen the knowledge and expertise of the local drug regulatory authorities in the developing world. The new generic prescribing initiative from the Drug Regulatory Authority of Pakistan is an excellent move, however, it cannot be ruled out that the risks of bribery and corruption may shift towards the pharmaceutical distributors or medical suppliers. In the absence of fully appreciating the underpinning science of drug discovery and regulatory compliance, the full benefits of the new prescribing regulations will be difficult to achieve. A similar attempt was made in the past through ‘The Drugs (Generic Names) Act in 1972’ and ‘The Drugs Act 1976’ in Pakistan but then failed to achieve its objectives. The national policy for drug registration, pricing, bioequivalence regulations, compendial compliance and SUPAC regulations are the main building blocks to establish the necessary infrastructure to successfully implement generic prescribing regulations in Pakistan and other low- and middle-income countries.
